# Supplementary material for: Conditional chemoconnectomics (cCCTomics) as a strategy for efficient and conditional targeting of chemical transmission
Source: eLife. 2024 Apr 30;12:RP91927. doi: 10.7554/eLife.91927 (PMC11060718; doi:10.7554/eLife.91927)
Supplement: Figure 3—source data 3. [file elife-91927-fig3-data3.zip › Figure 3 source data3 part 2.zip!/Figure 3-Source data 3-part2/1060f2-1-CRISPResso_Report_gl5umwyq.zip!/CRISPResso_on_1060f2_4/2b.Nucleotide_percentage_quilt_around_sgRNA_gcccacttcctggagtatcg.pdf]

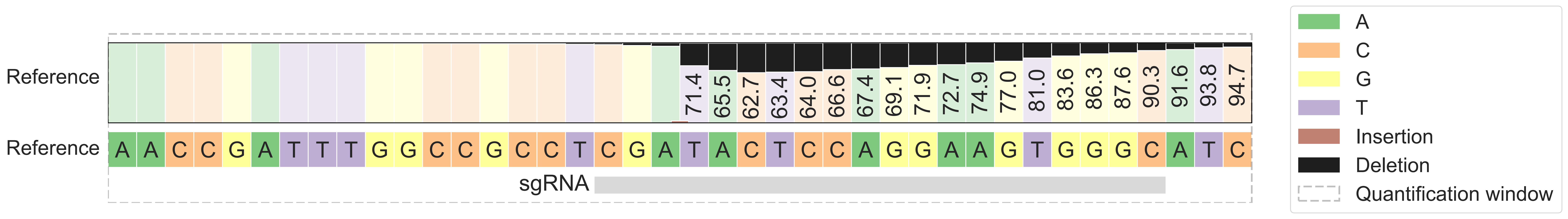

Reference
71.4
65.5
62.7
63.4
64.0
66.6
67.4
69.1
71.9
72.7
74.9
77.0
81.0
83.6
86.3
87.6
90.3
91.6
93.8
94.7
Reference
A A C C G A T T T G G C C G C C T C G A T A C T C C A G G A A G T G G G C A T C
sgRNA
A
C
G
T
Insertion
Deletion
Quantification window
